# Evaluation of the heart sounds in children using a Doppler Phonolyser

**DOI:** 10.1186/s12938-023-01084-0

**Published:** 2023-03-10

**Authors:** Mohammad Reza Khalilian, Mahsa Safari, Mahmoud Hajipour, Khosro Rahmani, Mahmoud Safari, Mohammad Hassan Ahmadpour, Tahmineh Tahouri

**Affiliations:** 1 Department of Pediatrics, Shahid Beheshti University of Medical Sciences, Shahid Modarres Educational Hospital, Intersection of Saadat Abad and Yadegar Imam Highway, Tehran, Iran; 2 Department of Pediatrics, Mofid Children’s Hospital, Shahid Beheshti University of Medical Sciences, Tehran, Iran; 3 Pediatric Gastroenterology, Hepatology and Nutrition Research Center, Research Institute for Children’s Health, Shahid Beheshti University of Medical Sciences, Tehran, Iran; 4 Head of Rheumatology Department Mofid Children’s Hospital, Shahid Beheshti University of Medical Sciences, Tehran, Iran; 5 Department of Anatomy, School of Medicine, Tehran University of Medical Sciences, Tehran, Iran; 6 Department of Nursing, Faculty of Nursing and Midwifery, Branch of Varamin and Pishva, Islamic Azad University, Tehran, Iran; 7 Pediatric Cardiology, Shahid Modarres Educational Hospital, Shahid Beheshti University of Medical Science, School of Medicine, Tehran, Iran

**Keywords:** Heart sound, Heart auscultation, Cardiac murmur, Auscultation, Innocent murmur, Congenital heart defects

## Abstract

**Background:**

Heart auscultation is an easy and inexpensive tool for early diagnosis of congenital heart defects. In this regard, a simple device which can be used easily by physicians for heart murmur detection will be very useful. The current study was conducted to evaluate the validity of a Doppler-based device named “Doppler Phonolyser” for the diagnosis of structural heart diseases in pediatric patients. In this cross-sectional study, 1272 patients under 16 years who were referred between April 2021 and February 2022, to a pediatric cardiology clinic in Mofid Children Hospital, Tehran, Iran, were enrolled. All the patients were examined by a single experienced pediatric cardiologist using a conventional stethoscope at the first step and a Doppler Phonolyser device at the second step. Afterward, the patient underwent trans-thoracic echocardiography, and the echocardiogram results were compared with the conventional stethoscope as well as the Doppler Phonolyser findings.

**Results:**

Sensitivity of the Doppler Phonolyser for detecting congenital heart defects was 90.5%. The specificity of the Doppler Phonolyser in detecting heart disease was 68.9% in compared with the specificity of the conventional stethoscope, which was 94.8%. Among the most common congenital heart defects in our study population, the sensitivity of the Doppler Phonolyser was 100% for detection of tetralogy of Fallot (TOF); In contrast, sensitivity of both the conventional stethoscope and the Doppler Phonolyser was relatively low for detecting atrial septal defect.

**Conclusions:**

Doppler Phonolyser could be useful as a diagnostic tool for the detection of congenital heart defects. The main advantages of the Doppler Phonolyser over the conventional stethoscope are no need for operator experience, the ability to distinguish innocent murmurs from the pathologic ones and no effect of environmental sounds on the performance of the device.

## Background

Congenital heart diseases are the most common congenital anomalies and affect about 0.8% of newborns [1]. The diagnosis of congenital heart disease (CHD) in children is usually made by physical examination findings, such as O2 saturation or heart murmurs. Despite the possibility that severe congenital heart diseases can be found without any clinical features, CHDs are diagnosed in about one-quarter of children with heart murmurs on routine physical examination. [2]. Heart auscultation is an easy, quick, noninvasive and inexpensive tool for early diagnosis [3] of congestive heart failure, structural heart disease, arrhythmia, etc. [4].

In a healthy heart, during cardiac cycles, two sounds called S1 and S2 are audible. These sounds are made by the opening and closure of the heart valves and variation in intensity and duration of them can be a sign of cardiac diseases. S3 or the third heart sound (which is a low frequency) can be heard after S2 as ventricular gallop; however, in pregnant females, athletes and children it may be considered as a physiologic sound. The fourth heart sound or S4, which is heard just before S1 is always pathologic [5]. Heart murmurs are caused by the turbulence and blood flow across the intra-cardiac defect, orifices, vessels as well as opening and closure of the heart valves [6]. Accordingly, heart murmurs can be heard in both physiologic states as well as structural heart diseases and have different types and intensities [5]. The frequency for these normal heart sounds is usually between 20 to 150 Hz, but the frequency of the murmurs heard in the hearts with structural anomalies is higher and ranges between 100 and 1000 Hz [3].

The stethoscope is a device which was invented in 1816 by Rene Laënnec for cardiac auscultation [6]. This acoustic device transfers the heart sounds through a hollow tube from the chest surface to the physician’s ear. The basis of auscultation with a stethoscope is mainly on mechanical vibration from the body surface at the frequency range of sound, which is between 20 and 20,000 Hz; however, mechanical vibrations below the frequency range of 20 to 20,000 Hz, which are called “infrasonic”, cannot be heard by the human’s ear [4]. Despite the important role of the auscultation with the stethoscope in the diagnosis of cardiac disease, it has many fundamental limitations; first, analysis of the heart sounds depends on the experience, knowledge and skills of the physician [7]. Moreover, the presence of breathing or environmental sounds can interfere with auscultation [7]. In this regard, another very important issue is the innocent murmurs. Innocent murmurs are relatively common with the prevalence of approximately 50–90% in the pediatric population. Considering the low incidence of congenital heart defects as well as acquired heart disease in this population, determining which patients with heart murmurs need to be referred to a pediatric cardiologist seems difficult, especially when they are asymptomatic [8]. Accordingly, a simple device which can be used easily by physicians for heart murmur detection, which can also distinguish pathologic murmurs from innocent murmurs, will be very useful.

The current study was conducted to evaluate the validity of a Doppler-based device named “Phonolyser” for the diagnosis of structural heart diseases in pediatric patients in comparison to the conventional stethoscope. The accuracy of diagnosing congenital and structural heart diseases using Doppler Phonolyser is compared to that of the diagnoses made by the conventional stethoscope.

## Results

### Background characteristics of patients

The median age of the patients was 58.2 ± 51.2 months. Of the total of 1272 patients, 96 (7.6%) were under 1 month, 271 (21.5%) were between 2 and 12 months, 477 (37.5%) were between 13 and 72 months and 415 (33.0%) were older than 72 months. Of these patients, 696 (54.7%) were males and 696 (54.7%) were females. The median weight of the patients was 19.3 ± 15.2 kg (Table 1).

**Table 1:** Baseline characteristics of the patients

Variable	Total	Heart disease	
		Absent (*N* = 798)	Present (*N* = 474)
Age (month)	58.2 ± 51.2	58.2 ± 51.2	56.5 ± 49.8
Sex			
Male	696 (54.7%)	450 (56.4%)	246 (51.9%)
Female	576 (45.3%)	348 (43.6%)	228 (48.1%)
Weight (kg)	19.3 ± 15.2	19.5 ± 15.7	18.8 ± 14.3
Age group			
0–1 month	96 (7.6%)	57 (7.2%)	39 (8.4%)
2–12 months	271 (21.5%)	177 (22.3%)	94 (20.1%)
13–72 months	477 (37.5%)	305 (38.5%)	172 (36.8%)
> 72 months	415 (33.0%)	253 (32.0%)	162 (34.7%)

### Comparison between the conventional stethoscope with the Doppler Phonolyser

Sensitivity of the conventional stethoscope and the Doppler Phonolyser for detecting congenital heart defects were 94.7% and 90.5%, respectively. The specificity of the conventional stethoscope in detecting heart disease was 94.8% in compared with the specificity of the Doppler Phonolyser, which was 68.9%. The positive predictive value of the conventional stethoscope and the Doppler Phonolyser were 91.6% and 63.3%, respectively, while the negative predictive value was 96.8% for the conventional stethoscope and 92.4% for the Doppler Phonolyser. Positive predictive value is the possibility that persons with a positive test really have the disease and negative predictive value is the possibility that persons with a negative test really do not have the disease. The positive likelihood ratio was 18.23 for the conventional stethoscope and 2.9 for the Doppler Phonolyser, while the negative likelihood ratio for the conventional stethoscope and the Doppler Phonolyser were 0.055 and 0.13, respectively (Table 2). The positive likelihood ratio (LR+) is the possibility that a positive test is observed in a patient divided by the possibility that a positive test is observed in a person without a disease and the negative likelihood ratio (LR-), the possibility of a patient with a negative test having a disease divided by the possibility of a patient with a negative test to not having a disease.

**Table 2:** Comparison between the conventional stethoscope and the Doppler Phonolyser in detection of congenital heart defects

	Total		Hearing				Phonolyser			
	Hearing	Phonolyser	0–1 month	1–12 months	12–72 months	Up to 72	0–1 month	1–12 months	12–72 months	Up to 72
Sensitivity	94.7%	90.5%	84.6%	91.9%	96.4%	98.7%	66.6%	85.8%	96.9%	97.5%
Specificity	94.8%	68.9%	94.7%	96.2%	95.7%	94%	75.4%	69.4%	72.4%	63%
PPV	91.6%	63.3%	91.6%	92.8%	91%	98.7%	65%	60.3%	65.2%	97.5%
NPV	96.8%	92.4%	90%	95.6%	96.9%	99.1%	76%	96.9%	94%	97.5%
LR+	18.23	2.90	16.05	23.99	18.37	16.65	2.71	2.80	3.33	2.64
LR−	0.055	0.13	0.16	0.08	0.03	0.01	0.044	0.20	0.11	0.04

In analysis of the results based on the gender, sensitivity as well as specificity of the Doppler phonolyser was similar to the conventional stethoscope (Table 3).

**Table 3:** Comparison between the sensitivity and specificity of the conventional stethoscope and the Doppler Phonolyser in both male and female patients

	Hearing		Phonolyser	
	Female	Male	Female	Male
Sensitivity	95.5%	93.8%	89.8%	91.2%
Specificity	94.4%	95%	66.4%	72.1%
PPV	90.4%	93%	59.4%	68.2%
NPV	97.5%	95%	92.3%	92%
LR +	15.83	18.6	2.61	3.37
LR-	0.05	0.07	0.16	0.12

### Comparison between the conventional stethoscopes with the Doppler Phonolyser in detecting the most common congenital heart defects

Table 4 indicates the sensitivity and specificity as well as the PPV, NPV, LR+ and LR− of the conventional stethoscope and the Doppler Phonolyser in detection of the most common congenital heart defects in our study population. Among the most common congenital heart defects, the sensitivity of the conventional stethoscope as well as the Doppler Phonolyser was 100% for detection of tetralogy of Fallot (TOF). In contrast, sensitivity of both the conventional stethoscope and the Doppler Phonolyser was relatively low for detecting atrial septal defect (ASD), especially for small defects in which the sensitivity of the conventional stethoscope and Doppler Phonolyser were 43.4% and 22.6%, respectively.

**Table 4:** Sensitivity and specificity of the conventional stethoscope and the Doppler Phonolyser for detection of the most common congenital heart defects in our study population

	Hearing						Phonolyser					
	PDA	VSD	ASD	Small ASD	TOF	MR	PDA	VSD	ASD	Small ASD	TOF	MR
SENSE	100%	100%	74.2%	48.4%	100%	97.4%	94.1%	98.4%	46.8%	22.6%	100%	98.3%
SPE	95%	95%	95%	95%	95%	95%	69.1%	69.1%	69.1%	69.1%	69.1%	69.1%
PPV	45.9%	61.2%	53.5%	27.3%	20%	73.8%	11.6%	20.1%	10.5%	2.8%	3.9%	31.7%
NPV	100%	100%	97.9%	97.9%	100%	99.7%	99.6%	99.8%	94.3%	95.8%	100%	99.6%
LR+	19.92	19.92	14.77	9.63	18.79	19.4	3.04	3.018	1.51	0.73	3.23	3.17
LR−	0	0	0.27	0.54	0	0.02	0.08	0.023	0.77	1.12	0	0.02

## Discussion

Currently, the main tool which is commonly used for auscultation of heart is a stethoscope. This acoustic device transfers the heart’s sounds through a hollow tube from the chest surface to the physician’s ear. The mechanical vibrations of the heart structures which are transmitted to the chest wall and have frequencies between 20 to 20,000 Hz can be detected by a stethoscope. [4]. Despite the important role of the stethoscope in the screening of the congenital heart defects, it has some major limitations; lack of experience of the examining physician, the presence of environmental interfering sounds, low frequency heart sounds and relative inability to distinguish innocent murmurs, all are limitations of the stethoscope in detecting congenital heart defects [7].

To date, various studies have been conducted on the efficacy of computer-based systems for auscultation of the heart [1]. De Vos et al. reported a novel system for computer-based auscultation of the heart with sensitivity and specificity of 90% and 96.5% for detection of congenital heart defects in 163 pediatric patients [10]. Kelmenson et al. presented a new prototype electronic stethoscope which had great accuracy in detection of congenital heart defects [11]. Will et al. also introduced a radar system for detection of heart sounds [12].

In this study, we evaluated the sensitivity, specificity and efficacy of a Doppler Phonolyser for detection of congenital heart defects. Our results show that Doppler Phonolyser can be applied for the examination of the heart. The sensitivity of this device for detection of congenital heart defects is 90.5%, which is near to the sensitivity of the conventional stethoscope, which was 94.7%. However, in patients under 1 year, especially in neonates, the sensitivity of the Doppler Phonolyser is relatively low (85.9% for infancy and 66.7% for the neonatal period). The possible cause for this is the fact that we used a single 2 Hz probe in all patients, while for the evaluation of the heart using ultrasound methods, high frequency probes (8–12 Hz) have been recommended. The specificity of the Doppler Phonolyser for detection of congenital heart defects is compared with a conventional stethoscope was relatively low, which was 68.9% for the Doppler Phonolyser and 94.8% for the conventional stethoscope. An important reason for the lower specificity of the Doppler Phonolyser compared to the conventional stethoscope is that the Doppler Phonolyser was not able to distinguish the tricuspid regurgitation as well as the pulmonary regurgitation murmurs, which are considered physiologic, from pathologic ones. The positive likelihood ratio (LR +) was low for both the conventional stethoscope and the Doppler Phonolyser which is probably due to the low prevalence of congenital heart disease in the population.

Among the most common congenital heart defects observed in our patients who had abnormal echocardiography, tetralogy of Fallot was detected in 100% of affected patients by the Doppler Phonolyser. Regarding other common congenital heart defects in our study population (including ventricular septal defect, patent ductus arteriosus, mitral regurgitation), the sensitivity of the Doppler Phonolyser was close to the conventional stethoscope except for atrial septal defect.

The sensitivity of hearing by a conventional stethoscope as well as using a Doppler Phonolyser for detection of atrial septal defects was relatively low at 74.2%; however, the sensitivity of the Doppler Phonolyser was even lower ( 46.8%). For the small atrial septal defects, the sensitivities for both modalities were very low (48.4% and 22.58% for the conventional stethoscope and the Doppler Phonolyser, respectively). Theoretically, this could be due the fact that the abnormal auscultation finding in the patients with atrial septal defects are usually subtle and are commonly confused with the innocent murmurs; in contrast, high pitch murmurs caused by patent Ductus Arteriosus, ventricular septal defect and pulmonary stenosis in patients with tetralogy of Fallot anatomy are easily detected by human ear as well as the Doppler Phonolyser. On the other hand, the classic auscultatory finding of an atrial septal defect is a wide fixed S2 splitting which can be missed by the Doppler Phonolyser. Besides, the systolic ejection murmur heard in ASD, can only be heard in patients who have relatively significant left-to-right shunt, resulting in a significant increase in pulmonary flow and functional pulmonary stenosis. In patients with a small size ASD, since the left-to-right shunt is not significant, the murmur could be subtle or even absent, and therefore, the specificity of the Doppler Phonolyser is lower than the larger defects [13].

Due to the increasing development of new medical technologies, the entry of any new device into this field requires economic justification for allocating very limited resources to countries with unlimited and increasing health needs.

One of the very important indicators of having a new technology is the accessibility index. It means physical access. Due to its low price and small size, this device can be used in all offices and medical centers, small and large, and has a very effective role in screening for congenital heart diseases especially in low-income and remote areas, where it is not possible to use advanced echocardiography devices.

Second t accessibility index is the fact that due to the much higher tariff for using echocardiography, using this device with a tariff below one thousand and five hundreds Rials, which is less than $ 5 per day, provides an excellent accessibility index for the possibility of financing its use for families of children and infants. Given the prevalence of congenital heart defects (about 4.2 cases per 1000 live births), even general screening of newborns in maternity wards with this device, will be cost benefitial due to the reduction in the imposition of high treatment costs later in life. The economics of family and community health have a very high justification.

In the process of diagnosing congenital heart disease from birth, early detection by this device leads to the accumulation of effective years of life and an increase in Qaly coefficient (Quality Adjusted Life Year). Late diagnosis of congenital heart defects imposes very high costs on the family, health insurance and the health system. In contrast, early diagnosis causes a significant increase in the accumulation of effective years of life, life expectancy indicators, reduction of hospital costs, reduction of required days of hospitalization, preventive surgery, reduction of the number of days lost or the Daly coefficient (Disability Adjusted Life Year), among other indicators. It is worth mentioning this device.

Other indicators are as follows: small size, low price, portability, the possibility of using the device in the least physical space, low maintenance costs, low capital depreciation, high cost efficiency and the possibility of using this device in maternity wards and neonatal wards without the need to move the patient to echocardiography, and also easy access of low-income people to this device, whether they are covered by health insurance or not, the possibility of easy movement of this device, management and reduction of hospital or treatment costs, etc.

It is recommended that a study be conducted on the effect of the Doppler Phonolyser based on health economics indicators to make accurate calculations of the cost-effectiveness and cost–benefit of using this device in reducing the cost of public health.

### Limitations

One of the limitations of this study was that auscultation with stethoscope was performed by an experienced pediatric cardiologist; it is obvious that the sensitivity and specificity of auscultation with a stethoscope will be affected when it is performed by a less experienced physician. The other limitation of this study was the fact that the Doppler Phonolyser device will not be able to detect cases of congenital heart diseases which do not cause a murmur (such as bicuspid aortic valve, patent foramen ovale, etc.) On the other hand, murmurs caused by physiologic mild tricuspid or pulmonary regurgitation, can be misdiagnosed by the Doppler Phonolyser device as a heart defect. Therefore, it seems that echocardiography is still the gold standard for the detection of congenital heart diseases and the Doppler Phonolyser device, mainly helps in diagnosing these defects in cases, where echocardiography is not available.

## Conclusion

This study evaluated the sensitivity, specificity and efficacy of a Doppler Phonolyser for detection of congenital heart defects. The main advantages of the Doppler Phonolyser over the conventional stethoscope are no need for operator experience, the ability to distinguish innocent murmurs from the pathologic ones and no effect of environmental sounds on the performance of the device. In overall, the Doppler Phonolyser could be useful as a diagnostic tool for detecting most congenital heart defects with sensitivity close to that of a conventional stethoscope. However, for detection of atrial septal defects is relatively low in compared with the conventional stethoscope. On the other hand, the specificity of the Doppler Phonolyser was lower than the conventional stethoscope. Future refinements of the device to increase its sensitivity and specificity may lead to an increase in the efficiency of the Doppler Phonolyser in detection of congenital heart defects.

## Materials and methods

### Doppler Phonolyser characteristics

**Fig. 1:**
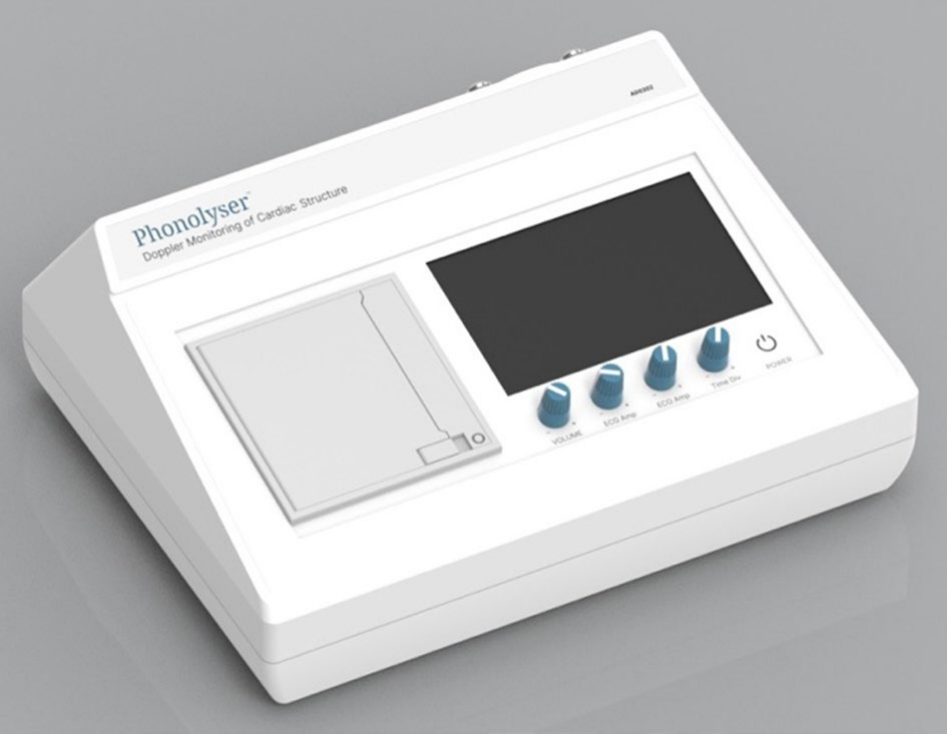
Doppler Phonolyser device

The Doppler Phonolyser AD0302 (Bu-Ali Research Institute, Mashhad, Iran, www.phonolyser.com) is a “smart heart sound analyzer based on the Doppler Effect” used to diagnose congenital and structural diseases of the heart (Fig. 1). Doppler, sound and electrocardiogram signals are displayed on the monitor of the device online synchronously. By this technique, the physician can determine the time of the murmurs. This device separates normal sound from a murmur by analyzing the heart sound. The physician can determine the time of the murmurs using the synchronization of the Doppler signal and ECG. The lack of influence of ambient noise and use of the Doppler Effect make the device more efficient in detecting cardiac murmurs. The Doppler Phonolyser overcomes two of the following technical issues that have long been of interest to physicians:

1. The Doppler Phonolyser shows a low gradient difference that cannot be heard through a stethoscope.
2. Cases such as ambient sounds, patient breathing, obesity and slimming and transpiration do not affect Doppler Phonolyser's function.

**Fig. 2:**
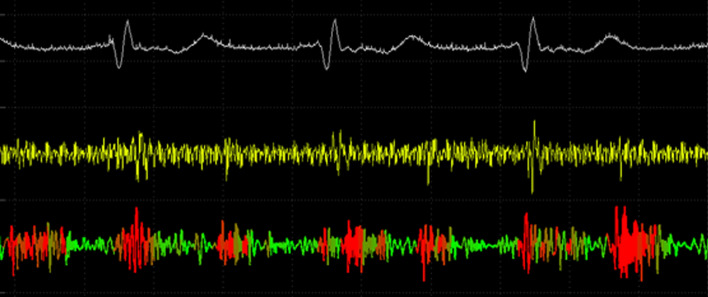
Doppler Phonolyser shows 3 graphs. the top graph shows the electrocardiogram signal, the middle graph shows the sound of the heart, and the bottom graph shows Doppler results

Phonolyser’s software is an AI-based software that detects abnormalities in the heart’s blood flow. It shows 3 graphs (Fig. 2).

The top graph is ECG that is used to find the systole and diastole time of the heart. The middle one shows the sound of the heart, and the bottom graph shows doppler results. If the heart has a normal structure the Doppler graph will be green and if due to congenital heart diseases the blood flow has any turbulence, the graph’s color will be changed to red.

The technical parameters are shown in Table 5.

**Table 5:** Technical parameters of Doppler Phonolyser

Parameters	Description
Prob	Ultrasound 2 MHz
Device diameters	275 × 204x97mm
power	100–240 V 50–60 Hz single-phase supply
LCD	5 inches resolution: 800 × 480
Printer	Thermal printer Paper width: 57.5 ± 0.5 mm paper roll diameter: 50 mm Max
Method(s) of sterilization	By methods validated and described by the manufacture
Suitability for use in an oxygen rich environment	Non-inclusion
Mode of operation	Continuous operation
Temperature	+ 10^C–+ 40^C
Humidity	< 80%
Pressure	86 kPa–106 kPa
Protection against harmful ingress of water or particulate matter	No production (IP00)

### Study design and study population

In this cross-sectional study, 1272 pediatric patients under 16 years who were referred for follow-up of a known congenital heart defect (before or after a corrective surgery) or for evaluation of a possible congenital heart disease between April 2021 and February 2022, to a pediatric cardiology clinic in Mofid Children Hospital, Tehran, Iran were enrolled. Characteristics of the patients, including their medical histories and diagnosis, were obtained from the parents and if needed from the electronic medical record system of our center. All the patients were examined by a single experienced pediatric cardiologist using a conventional stethoscope at the first step and a Doppler Phonolyser device at the second step. In this regard, while the patient was in the supine position, an Ultrasound 2 MHz probe of a Doppler Phonolyser device was firmly secured on the chest for 30 s in each of four usual auscultatory areas. The Doppler Phonolyser’s results were interpreted based on the Doppler graph (Fig. 2). A checklist in which the patients were classified based on the auscultation findings as well as Doppler Phonolyser findings in three groups (normal, innocent murmur and pathologic murmur) was completed. A second pediatric cardiologist blindly re-examined 120 patients of the total patients with the Doppler Phonolyser device and the findings were recorded in a second checklist. Afterward, the patient underwent trans-thoracic echocardiography with either a GE Vivid S60 or a Samsung HS70 echocardiographic system. The echocardiogram was interpreted without the knowledge of Doppler Phonolyser results. The echocardiogram was considered normal if there was no pathologic finding other than mild tricuspid or pulmonary regurgitation.

Consent for participation was obtained from the parents of the participants and the protocol was conducted in compliance with the Declaration of Helsinki and approved by the Ethical committee of Shahid Beheshti University of Medical Science (Ethics approval number: IR.SBMU.MSP.REC.1400.641).

### Statistical analysis

The categorical variables were calculated as counts and percentages and the continuous variables were calculated as mean (SD). The main goal of this study was the diagnostic accuracy of the Doppler Phonolyser device for detecting congenital heart defects in comparison with the conventional stethoscope. To evaluate the validity, the sensitivity, specificity, positive and negative likelihood and positive and negative predictive values for both the conventional stethoscope as well as the hearing by a conventional stethoscope were calculated.

Inter-rater agreement is the degree of agreement among observers who are assessing a same phenomenon and the Kappa statistic is usually used to measure inter-rater reliability for categorical items. When the raters are in full agreement, then Kappa = 1, and when there is no agreement between the raters, then Kappa = 0. To measure Intra subject Reliability, one group of patients was checked twice using the Doppler Phonolyser device, and the kappa value (*k* = 0.66) was reported which indicates substantial agreement. Afterward, inter subject reliability was investigated with the Doppler Phonolyser device in two groups of the patients and the Kappa value (*k* = 0.81) was reported, which indicates almost perfect agreement. To measure the intra-observer reliability, the results of the Doppler Phonolyser device were checked and interpreted twice by a pediatric cardiologist, and the Kappa value (*k* = 0.83) was reported which shows almost perfect agreement. To measure the inter observer reliability, the results of the Doppler Phonolyser device were examined by two expertized pediatric cardiologist separately and blinded, and the Kappa value (*k* = 0.71) was obtained, which indicates substantial agreement. For the statistical analysis, IBM SPSS Statistics 24for Windows (IBM Inc., Armonk, NY) was used.

## Data Availability

All data generated or analysed during this study are included in this published article (and its additional files).
